# A study on the correlation between the mean platelet volume to monocyte count ratio and long-term prognosis in patients with newly diagnosed coronary artery disease

**DOI:** 10.3389/fcvm.2025.1643542

**Published:** 2025-10-09

**Authors:** Wei Fu, Honghou He, Jianan Xu, Peihong Wu, Qian Zhang, Mei Wei, Linan Duan, Gang Wang, Le Wang, Zelong Cao, Mingqi Zheng

**Affiliations:** 1 Department of Cardiology, The First Hospital of Hebei Medical University, Shijiazhuang, Hebei, China; 2 Hebei Key Laboratory of Heart and Metabolism, Shijiazhuang, Hebei, China

**Keywords:** mean platelet volume, monocyte count, cardiovascular risk, major adverse cardiovascular events, the mean platelet volume/monocyte ratio

## Abstract

**Background:**

Coronary atherosclerotic heart disease (CAD) remains a major global health burden and a leading cause of mortality. Its pathogenesis is closely linked to multiple risk factors, among which inflammation plays a central role. While inflammatory biomarkers such as platelet and monocyte counts have been incorporated into prognostic assessments, their predictive accuracy remains limited. Further investigation of novel inflammatory indices is needed to refine risk stratification and guide clinical management.

**Objective:**

This study aimed to evaluate the prognostic value of the mean platelet volume-to-monocyte count ratio (MMR) for predicting major adverse cardiovascular events (MACE) in patients with newly diagnosed CAD.

**Methods:**

A total of 652 treatment-naïve CAD patients were enrolled. Kaplan–Meier survival analysis and univariate Cox proportional hazards models were applied to assess the association between MMR levels and MACE. Subgroup analyses were performed to test for effect modification. Restricted cubic spline (RCS) models were used to explore the dose–response relationship. The incremental predictive value of MMR beyond conventional risk factors was examined using changes in the concordance index (C-index), net reclassification improvement (NRI), and integrated discrimination improvement (IDI).

**Results:**

Patients were stratified into quintiles based on MMR values (L1: 7.89–14.43; L2: 14.50–17.96; L3: 18.00–22.16; L4: 22.25–28.53; L5: 28.67–60.67). Kaplan–Meier analysis revealed significantly poorer outcomes in the L3 group compared with other quintiles (log-rank *P* = 0.0014). RCS analysis demonstrated a significant nonlinear association between MMR levels and MACE risk (*P* = 0.001), characterized by an inverted U-shaped relationship. Incorporating MMR into conventional risk models significantly improved predictive performance (AUC 0.718 vs. 0.673; *P* = 0.018).

**Conclusion:**

In newly diagnosed CAD patients, MMR shows a nonlinear, inverted U-shaped association with MACE risk. The addition of MMR to standard risk models enhances prognostic accuracy. Further multicenter prospective studies and mechanistic trials are needed to verify the prognostic value of MMR and to elucidate its mechanism of action.

## 1 Introduction

The prevalence of coronary artery disease (CAD) has increased significantly. This situation now poses a serious public health threat, endangering population health and having a major global impact (1). CAD pathogenesis involves multifactorial processes (2). Atherosclerosis serves as its primary pathological basis, with complex mechanisms driving progression (3, 4). Inflammatory responses are pivotal in coronary atherosclerosis, where platelet activation and monocyte recruitment/differentiation crucially modulate plaque formation and evolution (5, 6). Upon endothelial injury, platelets adhere to exposed subendothelial matrices and release inflammatory mediators/chemokines, facilitating monocyte adhesion (7, 8). Chemotactic gradients then drive monocytes to infiltrate the intima, polarize into M1 macrophages, phagocytose oxidized lipids, and transform into foam cells—accelerating plaque progression (9). Conversely, when platelet activity is low, macrophages polarize toward the M2 phenotype. M2 macrophages suppress fibrous cap degradation, enhance plaque stability, reduce rupture risk, and prevent thrombosis (10, 11). This evidence indicates a dynamic balance between platelet activity and monocyte function in modulating plaque pathogenesis (12).

Atherosclerosis is recognized as an inflammatory disease, with immune dysregulation playing a central role. Inflammation permeates all stages of atherosclerosis, spurring interest in inflammatory biomarkers (13). Indices like systemic immune-inflammation index (SII), neutrophil-to-lymphocyte ratio (NLR), and platelet-to-lymphocyte ratio (PLR) correlate with CAD (14). Although existing biomarkers show prognostic utility (15), their clinical application remains suboptimal due to susceptibility to confounding variables, leading to inconsistent findings (16, 17). Consequently, novel inflammatory indices are needed to enhance prognostic accuracy, alleviate patient burden, and optimize clinical decision-making (18).

The mean platelet volume-to-monocyte count ratio (MMR), an inflammatory index previously linked to chronic obstructive pulmonary disease (COPD) phenotyping (19), has not been investigated in CAD. This study aimed to evaluate MMR's prognostic value in treatment-naïve CAD patients. We hypothesized that integrating MMR would augment traditional models' predictive capacity for CAD outcomes.

## 2 Methods

### 2.1 Study design and population

This study enrolled patients who underwent coronary angiography at the First Hospital of Hebei Medical University between August 1, 2018, and March 30, 2020. Eligible participants were newly diagnosed with coronary artery disease (CAD) and had not received prior treatment. All participants provided informed consent for the anonymous use of their clinical data. Exclusion criteria were: angiographic stenosis <50%, confirmed infectious disease, stage 5 chronic kidney disease, heart failure, non-ischemic cardiac conditions (e.g., severe valvular disease, acute myocarditis, malignant arrhythmias of non-ischemic origin, primary dilated cardiomyopathy, hypertrophic cardiomyopathy), suspected malignancy, Conn's syndrome, Cushing's syndrome, hypothyroidism, and incomplete clinical data. These criteria were applied to ensure a homogeneous cohort and to minimize confounding factors affecting inflammatory markers or cardiovascular outcomes. This study was approved by the Ethics Committee of the First Hospital of Hebei Medical University (ethical approval number: 20220362), and conducted in line with the Declaration of Helsinki.

### 2.2 Follow-up and endpoints

**Figure 1:**
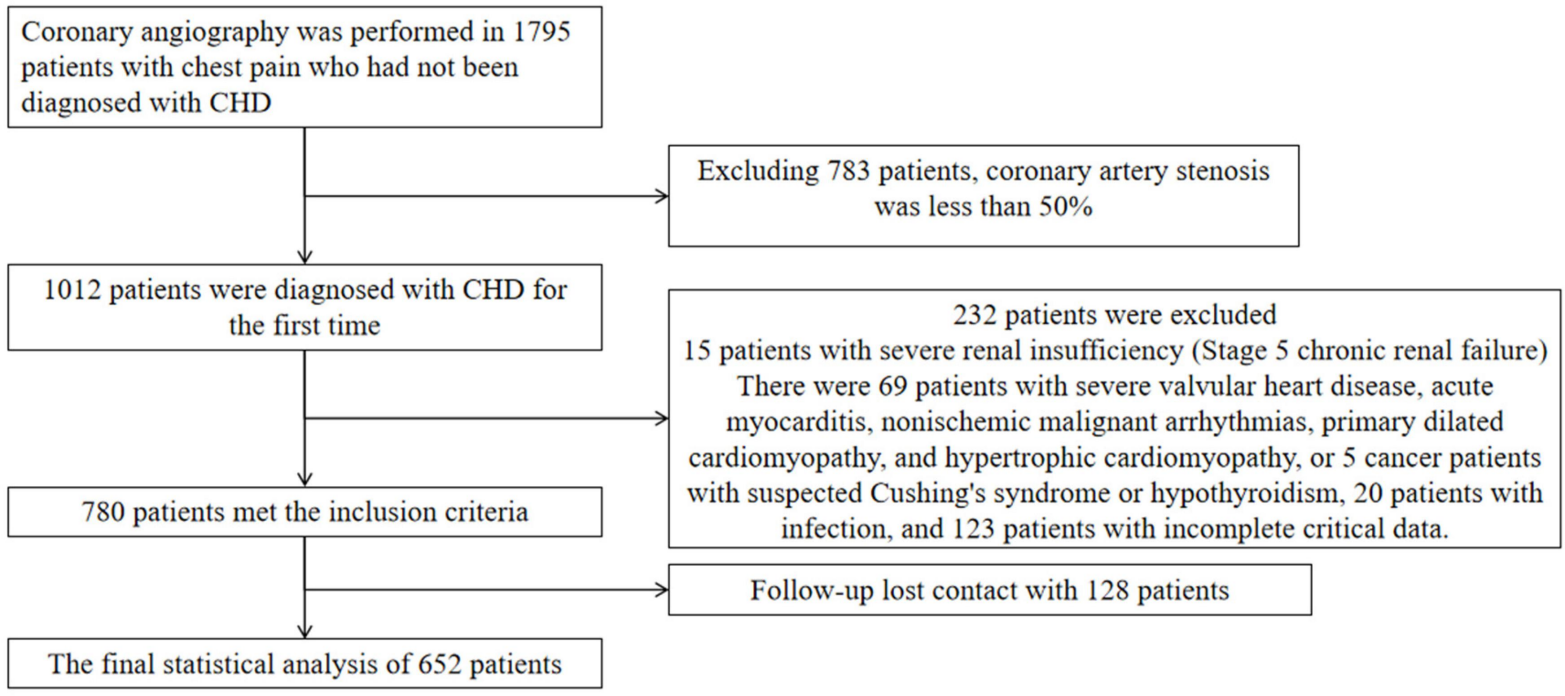
Flowchart of the study participants.

Patients were followed up through outpatient visits or telephone interviews at 1 month, 3 months, 6 months, 1 year, and annually thereafter for up to 5 years. Follow-up data were supplemented and verified using electronic health records. The primary endpoint was major adverse cardiovascular events (MACE), defined as all-cause mortality, nonfatal myocardial infarction, reperfusion therapy, stroke, and readmission for heart failure or severe angina. For the purposes of this study, all-cause mortality, nonfatal myocardial infarction, reperfusion, and stroke were considered MACE(hard endpoints). A total of 652 patients were included in the final analysis (Figure 1).

### 2.3 Baseline data collection

Baseline demographic and clinical data were collected at admission, including age, sex, body mass index (BMI), smoking status, alcohol consumption, and medical history (hypertension, diabetes, and cardiovascular disease). Laboratory tests were performed on fasting blood samples obtained within 24 h of admission, including total cholesterol (TC), low-density lipoprotein cholesterol (LDL-C), high-density lipoprotein cholesterol (HDL-C), triglycerides (TG), estimated glomerular filtration rate (eGFR), blood urea nitrogen (BUN), mean platelet volume, and monocyte count.

### 2.4 Assessment of anatomical stenosis severity in coronary artery disease

All patients underwent coronary angiography (CAG), and the severity of coronary stenosis was quantified using the Gensini scoring system. This system assigns points based on the degree of luminal narrowing (<25% = 1 point; 25%–49% = 2; 50%–74% = 4; 75%–89% = 8; 90%–98% = 16; total occlusion = 32), which are then multiplied by vessel-specific weighting factors (e.g., left main ×5; proximal left anterior descending ×2.5; mid ×1.5; distal ×1; diagonal branches ×1/0.5; proximal left circumflex ×2.5; distal or posterior descending ×1; posterolateral ×0.5; right coronary segments ×1). The total Gensini score was calculated as the sum of all lesion-specific scores. Assessments were independently reviewed by two board-certified cardiologists, and discrepancies were resolved by consensus.

### 2.5 Statistical analysis

Statistical analyses were performed using SPSS version 27.0 (IBM Corp., Armonk, NY, USA) and R version 4.2.3 (R Foundation for Statistical Computing, Vienna, Austria). Baseline characteristics were compared across quintiles of MMR. Categorical variables are presented as *n* (%), and continuous variables as mean ± standard deviation or median (interquartile range), depending on distribution. Normality was assessed using the Kolmogorov–Smirnov test, and variance homogeneity with Levene's test. Group differences were analyzed using the chi-square test for categorical variables, one-way ANOVA for normally distributed continuous variables, and the Kruskal–Wallis test for non-normally distributed variables.

Kaplan–Meier survival curves with log-rank tests were used to compare event-free survival across MMR groups. Cox proportional hazards models were applied to estimate hazard ratios (HRs) and 95% confidence intervals (CIs) for MACE, adjusting for clinically relevant confounders and variables significant in univariate analysis. Predictive performance was further assessed using receiver operating characteristic (ROC) curves. Subgroup analyses were conducted by sex, age, hypertension, and diabetes status, with interaction terms tested for effect modification. Restricted cubic spline (RCS) regression was used to evaluate potential nonlinear associations between MMR and MACE risk. A two-sided *P* value < 0.05 was considered statistically significant.

## 3 Results

### 3.1 Baseline characteristics

A total of 652 patients were enrolled, including 222 women (34.0%), with a mean age of 60.8 years. Among them, 377 (57.8%) presented with angina pectoris, 181 (27.3%) had diabetes mellitus, and 399 (60.0%) had hypertension. The mean MMR was 21.8. Patients were stratified into quintiles by MMR values: L1 (*n* = 131; 7.89–14.43), L2 (*n* = 128; 14.50–17.96), L3 (*n* = 131; 18.00–22.16), L4 (*n* = 131; 22.25–28.53), and L5 (*n* = 131; 28.67–60.67). Significant differences in several clinical variables were observed across quintiles, whereas age, creatinine, blood urea nitrogen, body mass index, estimated glomerular filtration rate, and cholesterol levels showed no significant variation (Table 1).

**Table 1:** Clinical characteristics of individual MMR level.

Value of the ratio of mean platelet volume to monocyte count						
Baseline variables	L1(*n*=131) 7.89–14.43	L2(*n*=128) 14.5–17.96	L3(*n*=131) 18–22.16	L4(*n*=131) 22.25–28.53	L5(*n*=131) 28.67–60.67	*P*
Age,years	62 (52,79)	61 (53,68)	62 (55,68)	63 (55,69)	60 (53,68)	0.381
Female, *n*(%)	26 (19.8)	31 (24.2)	47 (35.9)	58 (44.3)	60 (45.8)	<0.001
Diabetes, *n*(%)	31 (23.7)	33 (25.8)	39 (29.8)	43 (32.8)	35 (26.7)	0.501
Cerebrovascular disease, *n*(%)	23 (15.3)	18 (14.1)	22 (16.8)	23 (17.6)	17 (13.0)	0.839
Family history of CAD, *n*(%)	5 (3.8)	6 (4.7)	6 (4.6)	7 (5.3)	2 (1.5)	0.559
Smoke, *n*(%)	48 (36.6)	39 (30.5)	37 (28.2)	36 (27.5)	32 (24.4)	0.263
Drink, *n*(%)	36 (27.5)	34 (26.6)	36 (27.5)	22 (16.8)	22 (16.8)	0.048
BMI, kg/m^2^	26 (24,29)	26 (24,28)	26 (23,28)	25 (23,28)	26 (24,28)	0.145
HDL, mmol/L	0.94 (0.79,1.08)	0.95 (0.83,1.09)	0.99 (0.84,1.17)	0.98 (0.88,1.16)	1.07 (0.93,1.21)	<0.001
LDL, mmol/L	2.70 (2.28,3.23)	2.75 (2.27,3.21)	2.74 (2.21,3.18)	2.79 (2.28,3.43)	2.84 (2.34,3.34)	0.397
TG, mmol/L	1.37 (1.05,2.10)	1.42 (1.09,1.87)	1.40 (0.98,1.99)	1.42 (0.99,2.18)	1.37 (0.99,1.91)	0.739
CHOL, mmol/L	4.31 (3.6,4.96)	4.375 (3.75,4.9825)	4.3 (3.68,5.07)	4.64 (3.74,5.30)	4.62 (3.85,5.37)	0.170
Monocyte, 10^9^/L	0.7 (0.60,0.72)	0.50 (0.50,0.53)	0.40 (0.40,0.46)	0.36 (0.30,0.40)	0.30 (0.22,0.30)	<0.001
MPV, fl	8.2 (7.6,8.7)	8.4 (7.7,8.9)	8.4 (8.1,9.0)	8.9 (8.1,9.7)	9.3 (8.7,9.9)	<0.001
MMR	12.47 (11.29,13.33)	16.20 (15.4,17.19)	20.00 (19.00,21.00)	25.00 (23.50,27.00)	32.81 (30.33,38.46)	<0.001
eGFR, ml/min/1.73 m^2^	94.0 (83.0,101.0)	94.5 (86.0,102.0)	92.0 (81.0,99.0)	93.0 (80.0,101.0)	93.0 (84.0,102.0)	0.535
Cr, μmol/L	70.5 (63.7,82.3)	72.7 (63.9,82.0)	72.0 (59.7,81.1)	67.8 (60.1,76.6)	69.5 (59.3,80.4)	0.074
BUN, mmol/L	5.0 (4.27,6.32)	5.14 (4.25,6.14)	5.22 (4.26,6.51)	5.09 (4.11,6.17)	5.05 (4.14,6.05)	0.827
FPG, mmol/L	5.34 (4.78,6.70)	5.425 (4.65,7.08)	5.52 (4.96,6.73)	5.45 (4.81,6.47)	5.38 (4.84,7.01)	0.862
LM, *n*(%)	17 (13)	8 (6.3)	10 (7.6)	8 (6.1)	11 (8.4)	0.251
LAD, *n*(%)	105 (80.2)	102 (79.7)	102 (77.9)	102 (77.9)	105 (80.2)	0.980
LCX, *n*(%)	68 (51.9)	64 (50)	63 (48.1)	60 (45.8)	55 (42)	0.541
RCA, *n*(%)	73 (55.7)	65 (50.8)	73 (55.7)	61 (46.6)	68 (51.9)	0.546
Gensini	34 (16.0,64)	28 (10,48)	30 (12,56)	28 (10,60)	32 (10,54)	0.538
Hypertension, *n*(%)						0.397
No	51 (38.9)	45 (35.2)	52 (39.7)	42 (32.1)	63 (48.1)	
Grade1	18 (13.7)	18 (14.1)	21 (16)	16 (12.2)	8 (6.1)	
Grade2	15 (11.5)	18 (14.1)	13 (9.9)	21 (16)	13 (9.9)	
Grade3	47 (35.9)	47 (36.7)	45 (34.4)	52 (39.7)	47(35.9)	
Diagnosis, *n*(%)						0.009
1	71 (54.2)	57 (44.5)	53 (40.5)	43 (32.8)	51 (38.9)	
2	60 (45.8)	71 (55.5)	78 (59.5)	88 (67.2)	80 (61.1)	

BMI, body mass index; CHOL, Serum total cholesterol; LDL, low-density lipoprotein; HDL, high-density lipoprotein; TG, triglycerides; BUN, blood urea nitrogen; eGFR, estimate glomerular filtra-tion rate; MPV, Mean platelet volume; MMR,Mean platelet volume to monocyte ratio; Cr, Creatinine; Diagnosis 1 is acute myocardial infarction, and Diagnosis 2 is angina; FPG, Fasting plasma glucose.

### 3.2 MMR levels and risk of experiencing MACE

**Figure 2:**
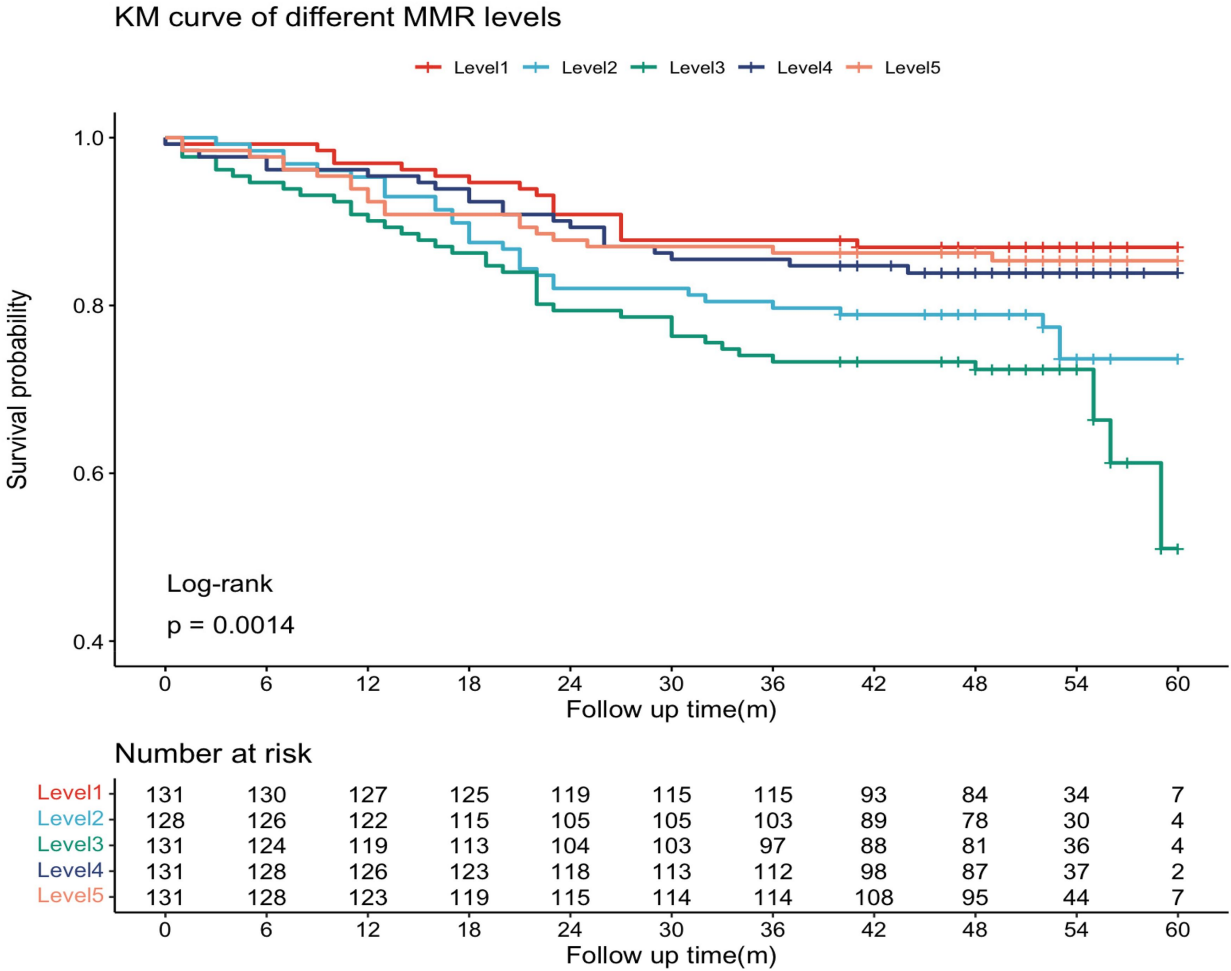
The Kaplan–Meier curves. The MMR was ranked from low to high, and the sample population was divided into five groups by quintile interval.

**Figure 3:**
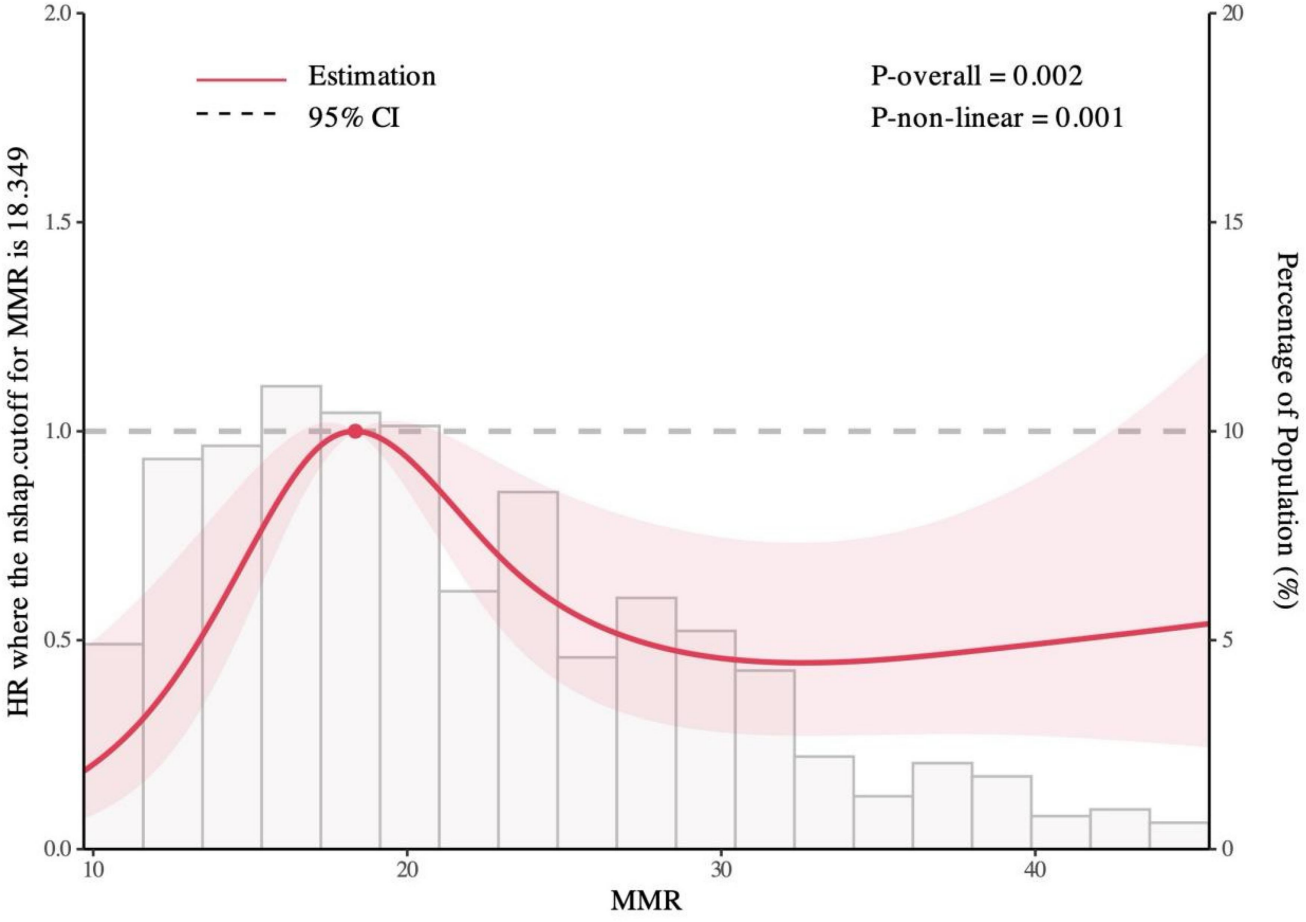
Restricted cubic spline plot from MMR levels vs. MACE.

The median follow-up duration was 51 months (IQR: 44 – 46 months). During follow-up, 127 patients (19.5%) experienced MACE, including 10 cardiovascular deaths (1.5%), 3 nonfatal myocardial infarctions (0.4%), 26 revascularizations (3.9%), and 102 rehospitalizations for heart failure or severe angina (15.6%). Kaplan–Meier survival curves by MMR quintiles are shown in Figure 2. Log-rank testing indicated significant differences in survival across groups (*P* = 0.0014), with patients in the L3 group exhibiting the poorest prognosis. Restricted cubic spline (RCS) analysis further demonstrated a significant nonlinear association between MMR levels and MACE risk (*P* = 0.001), characterized by an inverted U-shaped curve (Figure 3). The inflection point was identified at MMR = 18.35: below this level, higher MMR was associated with increased risk, whereas above this threshold, higher MMR predicted more favorable outcomes.

**Figure 4:**
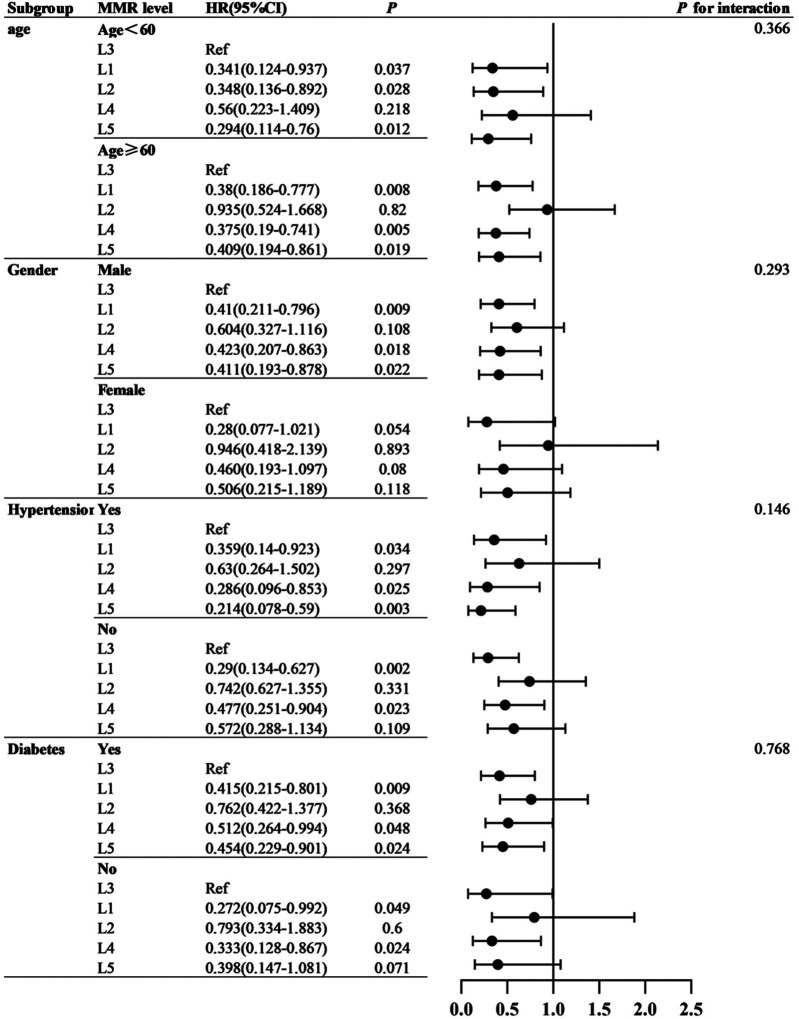
The relationship between MMR and the risk of MACE in different subgroups of patients.

The MMR values were sorted from low to high. The sample population was divided into five groups at quintile intervals: L1 (*n* = 131, MMR range 7.89–14.43), L2 (*n* = 128, MMR range 14.5–17.96), L3 (*n* = 131, MMR range 18–22.16), L4 (*n* = 131, MMR range 22.25–28.53), and L5 (*n* = 131, MMR range 28.67–60.67).

### 3.3 Independent association between MMR levels and risk of experiencing MACE

Cox proportional hazards analyses were performed to evaluate the association between MMR and MACE risk. Univariate Cox regression identified several baseline variables associated with MACE (Table 2). Predictive models incorporated covariates from established prognostic frameworks and variables with *P* < 0.10 in univariate analysis: Model 1 adjusted for age and sex; Model 2 additionally included diabetes, hypertension grade, CAD type, and Gensini score; Model 3 further incorporated eGFR, LDL-C, triglycerides, and fasting glucose. As shown in Table 3, patients in the L3 group had significantly higher risk of both MACE and hard endpoints compared with other quintiles. Specifically, L3 was independently associated with increased MACE risk across all models, with significant differences vs. L1, L4, and L5. Subgroup analyses demonstrated consistent results across sex, age, diabetes, and hypertension strata, with no evidence of significant interaction effects (all interaction *P* > 0.05). Refer to Figure 4.

**Table 2:** The Unadjusted hazard ratios of each indicator for the risk of MACE.

Factors	Risk of MACE			
	HR	95%CI		*P*
Age, years	1.019	1.002	1.038	0.330
Female, *n*(%)	1.110	0.773	1.594	0.571
Diabetes, *n*(%)	1.182	0.811	1.724	0.384
Cerebrovascular disease, *n*(%)	1.312	0.841	2.046	0.231
Family history of CAD, *n*(%)	1.445	0.674	3.098	0.344
Smoke, *n*(%)	0.910	0.617	1.344	0.637
Drink, *n*(%)	0.813	0.525	1.259	0.353
BMI, kg/m^2^	1.032	0.982	1.084	0.220
HDL, mmol/L	0.583	0.266	1.277	0.177
LDL, mmol/L	0.992	0.791	1.244	0.944
TG, mmol/L	1.174	1.010	1.366	0.037
CHOL, mmol/L	1.035	0.886	1.209	0.661
eGFR, ml/min/1.73 m^2^	0.990	0.981	1.000	0.043
Cr, μmol/L	1.001	0.996	1.006	0.714
BUN, mmol/L	1.033	0.944	1.130	0.479
FPG, mmol/L	1.130	1.062	1.201	0.001
LM, *n*(%)	2.122	1.303	3.457	0.003
LAD, *n*(%)	1.214	0.767	1.922	0.408
LCX, *n*(%)	1.310	0.925	1.857	0.129
RCA, *n*(%)	1.479	1.034	2.107	0.032
Gensini	1.009	1.005	1.013	0.010
Hypertension, *n*(%)	1.331	0.919	1.926	0.130
Diagnosis, *n*(%)	0.681	0.481	0.964	0.030

BMI, body mass index; CHOL, Serum total cholesterol; LDL, low-density lipoprotein; HDL, high-density lipoprotein; TG, triglycerides; BUN, blood urea nitrogen; eGFR, estimate glomerular filtra-tion rate; MPV, Mean platelet volume; MMR, Mean platelet volume to monocyte ratio; Cr, Creatinine; FPG, Fasting plasma glucose.

**Table 3:** The risk relationship of MMR with the occurrence of MACE and MACE (hard endpoints).

Event			Model 1		Model 2		Model 3	
			HR(95% CI)	*P*	HR(95% CI)	*P*	HR(95% CI)	*P*
MACE	A	L3	1.920 (1.320–2.793)	0.001	1.922 (1.320–2.798)	0.001	2.074 (1.415–3.041)	0.001
	B	L3	Ref		Ref		Ref	
		L1	0.396 (0.224–0.700)	0.001	0.376 (0.212–0.665)	0.001	0.354 (0.200–0.629)	0.001
		L2	0.775 (0.482–1.248)	0.294	0.799 (0.496–1.287)	0.356	0.733 (0.452–1.187)	0.206
		L4	0.484 (0.285–0.821)	0.007	0.473 (0.278–0.806)	0.006	0.441 (0.258–0.756)	0.003
		L5	0.454 (0.263–0.785)	0.005	0.475 (0.274–0.824)	0.008	0.430 (0.246–0.752)	0.003
MACE (hard endpoints)	A	L3	2.944 (1.612–5.375)	<0.001	2.872 (1.564–5.274)	0.001	2.916 (1.558–5.457)	0.001
	B	L3	Ref		Ref		Ref	
		L1	0.397 (0.171–0.919)	0.031	0.383 (0.165–0.888)	0.025	0.381 (0.163–0.892)	0.026
		L2	0.383 (0.159–0.921)	0.032	0.409 (0.170–0.986)	0.046	0.405 (0.165–0.992)	0.048
		L4	0.330 (0.131–0.834)	0.019	0.322 (0.127–0.821)	0.018	0.318 (0.123–0.820)	0.018
		L5	0.252 (0.093–0.681)	0.007	0.274 (0.100–0.749)	0.012	0.263(0.094–0.732)	0.011

MACE refers to all-cause mortality, non-fatal myocardial infarction, reperfusion therapy, stroke, and readmission due to heart failure or severe angina pectoris. MACE (hard endpoints) includes all-cause mortality, non-fatal myocardial infarction, reperfusion therapy and stroke. A refers to the Cox multivariate regression analysis using MMR L3; B refers to the multivariate Cox regression analysis of the MMR 5 group, with L3 serving as the control group. Model 1: Adjusted for age and gender. Model 2: Based on Model 1, adjusted for diabetes status, hypertension status (no hypertension, hypertension 1–3 grades), coronary artery disease type (angina pectoris or acute coronary syndrome), and severity of coronary artery disease (gensini score). Model 3: Further adjusted for eGFR, LDL-C, TG concentration, and fasting blood glucose.

### 3.4 Improvements in cardiovascular risk prediction

**Figure 5:**
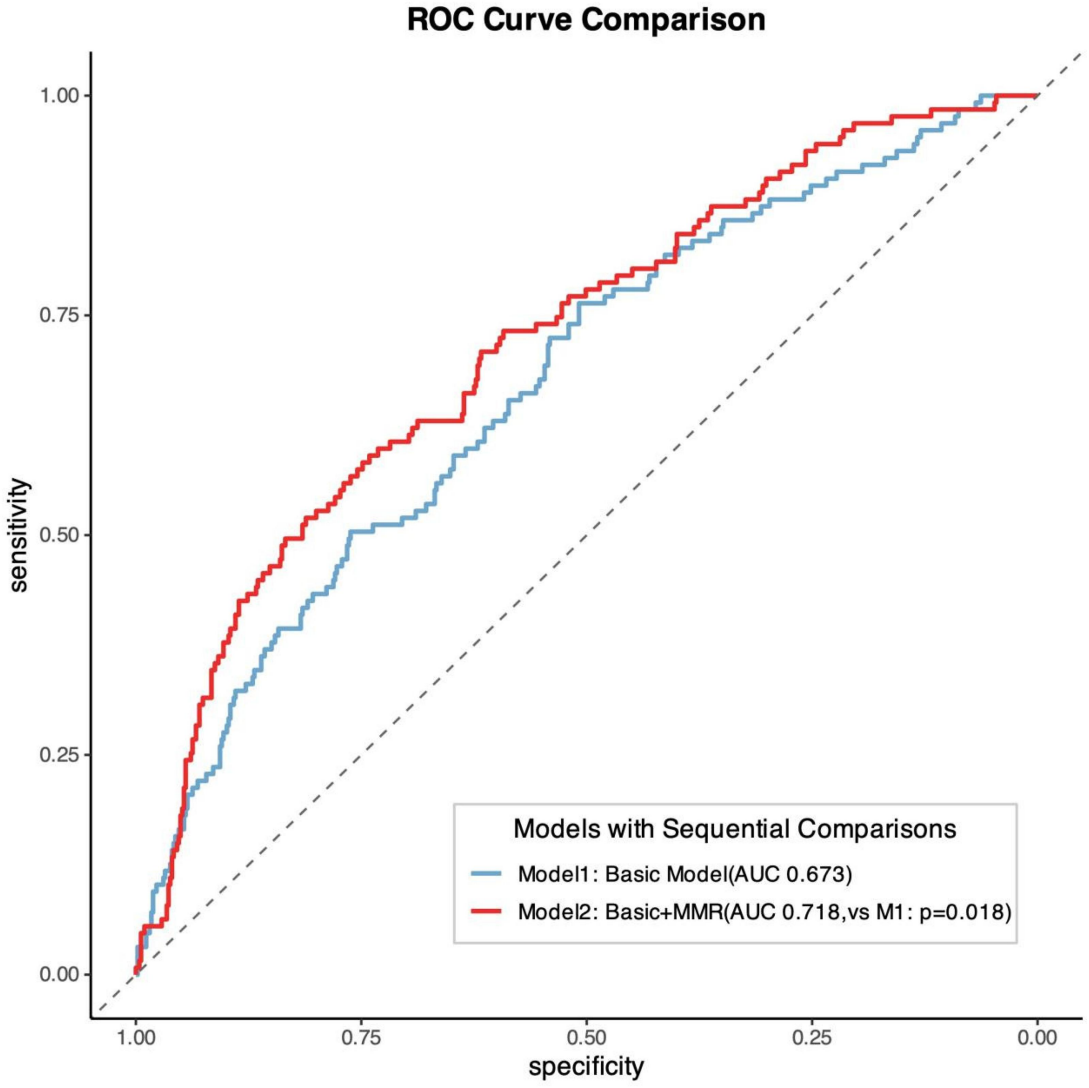
Comparison of ROC curves for predicting MACE by different models.

The addition of MMR quintiles to conventional cardiovascular risk models significantly improved prognostic performance. The baseline model, which included age, sex, hypertension grade, diabetes mellitus, CAD type, Gensini score, triglycerides, LDL-C, eGFR, and fasting plasma glucose, yielded a C-index of 0.657 and an AUC of 0.673. After incorporating MMR, the C-index increased to 0.691 (*P* = 0.035) and the AUC improved to 0.718 (*P* = 0.018). Moreover, both net reclassification improvement (NRI) and integrated discrimination improvement (IDI) were significantly enhanced (*P* < 0.001 for both; Figure 5 and Table 4).

**Table 4:** Improving the reclassification and discrimination capabilities of MACE risks based on MMR.

Analysis	BM (95% CI)	BM + MMR (95% CI)	Value/*Δ*	95% CI	*P*
C-index	0.66 (0.61–0.71)	0.69 (0.64–0.74)	–	–	0.035
AUC	0.67	0.72	–	–	0.018
NRI-Categorical	–	–	0.12	0.05–0.19	<0.001
NRI-Continuous	–	–	0.38	0.19–0.57	<0.001
IDI	–	–	0.04	0.02–0.05	<0.001

The basic model incorporates age, gender, hypertension classification, diabetes, gensini score, type of coronary heart disease, TG, LDL, eGFR, and FPG. Basic + MMR is a new model that adds MMR to the basic model.

The basic model incorporates age, gender, hypertension classification, diabetes, gensini score, type of coronary heart disease, TG, LDL, eGFR, and FPG. Basic + MMR is a new model that adds MMR to the basic model.

## 4 Discussion

This study evaluated the prognostic significance of MMR in treatment-naïve CAD patients. We found that patients in the intermediate MMR range (18.0–22.16) experienced the highest incidence of MACE, whereas both lower and higher MMR values were associated with more favorable outcomes. Unlike the linear associations typically reported for other inflammatory biomarkers, our restricted cubic spline analysis revealed a distinct nonlinear, inverted U-shaped relationship between MMR and long-term prognosis.

Previous studies have established inflammation as a key driver of atherosclerosis, with indices such as NLR, PLR, and MLR serving as independent prognostic markers in CAD (20, 21). Composite inflammatory indices show utility beyond CAD diagnosis, severity assessment, and prognosis prediction to oncology/hematological disorders (22, 23). However, clinical implementation faces limitations: (1) inconsistent findings regarding their independence as CAD risk predictors (15, 16); (2) substantial threshold variation for MACE prediction; (3) susceptibility to confounders (infections, medications, comorbidities) compromising validity (24); (4) inability to independently predict MACE, necessitating integration into multivariate models (25). Novel indices like systemic inflammation response index (SIRI) exhibit superior MACE predictive value vs. NLR/PLR/MLR. SIRI-incorporated models significantly enhance diagnostic performance (26). In the comparison of the clinical value of composite inflammatory markers and single inflammatory markers, we used MMR, MPV and monocyte count for statistical analysis. Multivariable Cox regression analyses, adjusted for age, gender, hypertension grade, diabetes mellitus, Gensini score, coronary heart disease type (angina pectoris or acute coronary syndrome), triglycerides (TG), LDL cholesterol, eGFR, and FPG, were performed to assess three incremental models: 1. Base model + MMR L3 status 2. Base model + Mean Platelet Volume (MPV) 3. Base model + absolute monocyte count Results demonstrated that MMR L3 was an independent risk factor for MACE (HR = 0.482; 95% CI: 0.329–0.707; *P* < 0.001). In contrast, neither MPV (HR = 1.066; 95% CI: 0.902–1.260; *P* = 0.454) nor absolute monocyte count (HR = 0.739; 95% CI: 0.243–2.245; *P* = 0.594) showed significant associations. This indicates that categorical MMR stratification (L3) demonstrates stronger predictive utility for MACE risk compared to the continuous variables MPV or monocyte count alone. So in some cases, a composite inflammatory measure is more clinically relevant than a single measure. Thus, discovering novel inflammatory biomarkers and refining predictive algorithms remains crucial for optimizing clinical decision-making. After adjusting for factors such as age, gender, history of diabetes and hypertension, coronary heart disease category (angina pectoris or acute myocardial infarction), eGFR, FPG, LDL, TG, and Gensini, we conducted a multivariate Cox regression analysis. Among them, Model a represents the MMR L3 group, Model b represents NLR, Model c represents PLR, and Model d represents MHR. Through the analysis, it was found that the MMR L3 group was an independent risk factor for MACE in newly diagnosed coronary heart disease patients (Model a HR = 2.07, *P* < 0.001), while the other indicators had no statistical significance (Model b, Model c, and Model d all had *P* values greater than 0.05). We can observe that the role value of MMR L3 in MACE in newly diagnosed coronary heart disease patients may be due to NLR, PLR, and MHR.

Our study revealed that patients in the MMR L3 quintile (18.0–22.16) exhibited the poorest prognosis compared to other groups. Restricted cubic spline (RCS) analysis confirmed a significant non-linear, inverted U-shaped relationship between MMR and MACE risk—a finding distinct from other composite inflammatory indices (27). The observed inverted U-shaped relationship may reflect a dynamic equilibrium between platelet activity and monocyte function in plaque biology. Platelets modulate monocyte adhesion, differentiation, and polarization. Under pro-inflammatory conditions (28), activated platelets promote M1 macrophage polarization, leading to collagen degradation, reactive oxygen species release, and plaque destabilization (29, 30). Platelet-derived SEMA4D induces M2 polarization (10). M2 macrophages release MMP inhibitors, secrete TGF-β to enhance vascular smooth muscle cell (VSMC) proliferation (strengthening fibrous caps) (31, 32), and produce IL-10 to suppress platelet activation. Platelet-monocyte coculture upregulates M2 markers (CD163) and scavenger receptors (SR-BI, CD36) (11). Notably, CAD patients' platelets exhibit elevated SR-BI/CD36 expression, promoting monocyte differentiation into atheroprotective M2 phenotypes. Additionally, platelet-monocyte aggregates (PMAs) serve as thromboinflammatory hubs through *P*-selectin/PSGL-1 binding → Mac-1/GPIbα-fibrinogen stabilization (33). This cascade drives plaque destabilization in acute coronary syndromes and restenosis, establishing PMAs as therapeutic targets (e.g., *P*-selectin inhibitors). Thus, an intermediate MMR range may reflect heightened pro-thrombotic and pro-inflammatory activity, whereas lower or higher MMR levels may favor protective M2-dominated pathways. This mechanistic hypothesis warrants validation through longitudinal and experimental studies.

Our findings indicate that both low and high MMR ranges confer better prognosis compared to the intermediate L3 quintile (18.0–22.16), potentially due to attenuated platelet activity and milder inflammatory responses that stabilize plaques and suppress thrombosis (34, 35). Conversely, MMR values within the 18.0–22.16 range may promote thrombogenesis and plaque vulnerability, worsening clinical outcomes. Analogous to sepsis and severe burns (36), this biphasic pattern suggests bidirectional inflammatory modulation in CAD progression: during early and late disease stages, protective mechanisms (e.g., M2 macrophage polarization, anti-inflammatory cytokine release) may outweigh pathological processes, whereas intermediate phases exhibit dominant pro-thrombotic and pro-inflammatory drivers. This dynamic homeostatic regulation could explain the inverted U-shaped risk curve. However, these hypotheses require validation, as the intricate relationship between platelet indices and monocyte biology remains incompletely characterized. In the future, by repeatedly measuring indicators such as MPV and absolute values of monocytes in patients during the occurrence and development of coronary heart disease, and studying the dynamic changes of MMR and its prognostic relationship with MACE, the possibility of this hypothesis can be tested. Further mechanistic studies are essential to elucidate these interactions, clarify our observations, and optimize translational applications for precision prognostication in CAD management.

Subgroup analyses by age, sex, diabetes, and hypertension status revealed consistent associations, with no significant interaction effects. Although some subgroup estimates did not reach statistical significance, the directionality was consistent with the overall findings. These results suggest the robustness of MMR as a prognostic marker, though larger cohorts are needed to confirm subgroup-specific effects (37).

The addition of MMR to conventional risk models significantly improved prognostic performance, as reflected by higher C-index, AUC, NRI, and IDI values. This supports the role of MMR as an incremental biomarker that enhances existing prognostic frameworks for CAD risk stratification.

## 5 Limitations

Several limitations should be acknowledged. First, the single-center design with limited demographic diversity may reduce generalizability (38, 39). Second, although patients with overt infection were excluded, residual confounding from subclinical inflammatory states cannot be ruled out. Third, inter-laboratory variability in MPV measurement, and the lack of a universal reference range, may affect MMR reproducibility. Fourth, our analysis focused on baseline values; serial measurements may provide greater insight into dynamic changes in MMR and their prognostic implications. Finally, because hard endpoint events were relatively infrequent, we used a composite MACE definition that included soft endpoints, which may limit interpretability. Larger, multicenter studies with dedicated hard endpoint analyses are warranted (40).

## 6 Conclusion

In conclusion, MMR is a readily available inflammatory index derived from routine blood tests that demonstrates incremental prognostic value in newly diagnosed CAD patients. The nonlinear, inverted U-shaped association between MMR and MACE highlights the complex role of platelet–monocyte dynamics in atherosclerosis. Further multicenter prospective studies and mechanistic trials are needed to verify the prognostic value of MMR and to elucidate its mechanism of action.

## Data Availability

The original contributions presented in the study are included in the article/Supplementary Material, further inquiries can be directed to the corresponding authors.
