# Correction: A study on the correlation between the mean platelet volume to monocyte count ratio and long-term prognosis in patients with newly diagnosed coronary artery disease

**DOI:** 10.3389/fcvm.2025.1721709

**Published:** 2025-11-11

**Authors:** Wei Fu, Honghou He, Jianan Xu, Peihong Wu, Qian Zhang, Mei Wei, Linan Duan, Gang Wang, Le Wang, Zelong Cao, Mingqi Zheng

**Affiliations:** 1 Department of Cardiology, The First Hospital of Hebei Medical University, Shijiazhuang, Hebei, China; 2 Hebei Key Laboratory of Heart and Metabolism, Shijiazhuang, Hebei, China

**Keywords:** mean platelet volume, monocyte count, cardiovascular risk, major adverse cardiovascular events, the mean platelet volume/monocyte ratio

File **Data Sheet 1.xlsx** was erroneously published with the original version of this paper due to a file selection error during submission. The file has now been replaced. The original version of this article has been updated.

